# The impact of the COVID-19 pandemic on respiratory failure caused by respiratory viruses in children and adolescents

**DOI:** 10.3389/fped.2024.1392426

**Published:** 2024-08-15

**Authors:** Thiago Belem Gama, Alexandre A. Ferraro, Sandra E. Vieira

**Affiliations:** Department of Pediatrics, Faculdade de Medicina da Universidade de São Paulo, São Paulo, Brazil

**Keywords:** COVID-19, infant, children, adolescent, respiratory virus, acute respiratory failure

## Abstract

**Background:**

In addition to the direct impact of the coronavirus disease 2019 (COVID-19) pandemic on child/adolescent health, changes in infections caused by other viruses have been observed. Respiratory syncytial virus (RSV) and influenza are important agents of acute respiratory failure (ARF) in these age groups. This study presents an analysis of the influence of the pandemic on the seasonal and clinical patterns of ARF caused by RSV and influenza.

**Methods:**

A retrospective ecological study was performed. The data of individuals younger than 20 years who were hospitalized with ARF and who were diagnosed with RSV, influenza and severe acute respiratory syndrome coronavirus 2 (SARS-CoV-2) between 2019 and 2022 were analysed. The data were collected from the governmental system.

**Results:**

Among 367,136 individuals, the incidence of ARF increased annually. During the pandemic, the number of infected schoolchildren, adolescents, and nonwhite people; intensive care admissions; and mortality rates increased. Older age, SARS-CoV-2 infection, and residence in North Brazil/Northeast Brazil were associated with lower odds of intensive care unit admission but greater odds of death. Comorbidities were important risk factors for severe disease. There was a drastic reduction in the number of RSV and influenza infections, with a resurgence in 2021. After the resurgence in 2021, the number of influenza-related deaths remained above the 2019 level, which did not occur in 2022. After 2021, RSV infection was associated with greater odds of intensive care admission but not death.

**Conclusions:**

During the pandemic, older children, adolescents, and individuals with comorbidities were more vulnerable to ARF. There was a reduction in the prevalence and severity of RSV and influenza infections. After this reduction, a resurgence with an out-of-season pattern, but without higher odds of death than in the prepandemic year, was observed for both in 2022.

## Introduction

In March 2020, the World Health Organization declared the coronavirus disease 2019 (COVID-19) outbreak a global pandemic. Severe acute respiratory syndrome coronavirus 2 (SARS-CoV-2) was first identified in the city of Wuhan, China, in December 2019. Brazil was one of the most affected countries, with millions of cases and more than 700,000 deaths (1). SARS-CoV-2 has become an important cause of morbidity and mortality, especially in elderly individuals and patients with comorbidities, and it also affects young adults, adolescents, and children (2, 3). Lower respiratory tract infections are the leading cause of death in children under 5 years of age worldwide (4, 5). However, adolescence is a particular phase of development with characteristics that differentiate it from childhood and adulthood. Behavioural and physiological patterns in adolescence confer different vulnerabilities to infectious conditions, such as viral respiratory infections (6). Antoon et al. studied factors associated with the severity of COVID-19 and reported that among hospitalized children and adolescents, older children and adolescents had a lower risk of hospitalization but more severe illness when hospitalized (7).

In addition to the direct impact of the COVID-19 pandemic on child and adolescent health, changes in infections caused by other respiratory viruses, such as respiratory syncytial virus (RSV) and influenza, have been observed. These changes had a remarkable influence on the implementation of nonpharmacological preventive interventions (8, 9).

Nonpharmacological interventions include the practice of social distancing; the protective use of face masks and hand sanitizers; and the closure of schools, businesses, borders, and other nonessential services. Furthermore, interactions between different viruses during this period may also have promoted changes in the severity of subsequent or concomitant respiratory infections (10).

RSV and influenza are important causes of acute respiratory failure (ARF) in children and adolescents and occur with seasonal and predictable patterns, which also allows for the development of specific prophylactic measures such as passive and active immunization (11, 12). However, external factors such as epidemics caused by variants or other respiratory viruses can interfere with the epidemiology and severity of these infections (8, 9).

The study of the factors associated with the severity of respiratory virus infections during the COVID-19 pandemic offers an interesting opportunity to better understand changes in typical seasonal patterns of RSV and influenza infections as well as transmission and vulnerability to respiratory viruses, develop prophylactic measures and prepare health systems (13, 14).

The objective of this study was to analyse the effects of the COVID-19 pandemic on children and adolescents and its influence on the incidence and severity of ARF caused by RSV and influenza infections in Brazil (15).

## Methods

### Study design and population

We conducted a cross-sectional study of hospitalized patients with ARF aged between 0 and 20 years in Brazil who were registered in the Influenza Epidemiological Surveillance Information System from January 2019 to December 2022 and had aetiological laboratory confirmation (15). Patients with coinfections were included only in the descriptive analysis. This information system is a platform for the registration of patients with ARF among hospitalized individuals with flu-like syndrome (FLS). FLS is defined as an acute respiratory condition with at least two of the following signs and symptoms: fever, chills, sore throat, headache, cough, runny nose, and olfactory and taste disorders. In children younger than 2 years of age, the sudden onset of fever and respiratory symptoms in the absence of another specific diagnosis are also considered. An aetiological investigation of respiratory secretions was performed via indirect immunofluorescence and molecular methods (PCR and RT‒PCR) for influenza and RSV and via RT‒PCR, RT‒LAMP, and immunochromatography for SARS-CoV-2. The system is supplied with data from public and private networks and covers various aetiological agents. The registration of hospitalized patients with ARF is mandatory.

### Data analysis

The prevalence, aetiology, and demographic and clinical characteristics of ARF in the study period and the changes that occurred each year were analysed and compared to those reported in 2019. Comparative analyses of patients infected with influenza, RSV, or SARS-CoV-2 were performed. The age ranges for the subcategories were as follows: newborn (0–28 days), infant (29 days–23 months), preschooler (24–59 months), schoolchildren (5–10 years), and adolescent (11–20 years).

Patients with coinfections were excluded from all comparative analyses. The absolute frequencies and percentages of the data were calculated. The chi-square test was used to determine associations. A multilevel mixed effects generalized linear model was built to determine the crude and adjusted odds ratios for confounding factors (gender, age, ethnicity, and origin). The “other aetiologies” group, which corresponds to the sum of other aetiological agents and cases without identified aetiologies, was used as the reference group in the multilevel mixed-effects generalized linear model. We assumed that the municipality where the patient resided was a random effect. A significance value of *p* < 0.05 was set. Sigma Stata v.2.0 software (SPSS Inc., Chicago, USA) was used.

## Results

### Clinical and demographic characteristics of patients with acute respiratory failure

A total of 367,136 patients with ARF were analysed. The demographic and clinical characteristics of the patients are presented in Table 1. The number of patients with ARF increased each year, reaching 27,397 in 2019, 88,998 in 2020, 150,403 in 2021, and 167,884 in 2022. In 2020 and 2022, there were significant increases in the percentages of infected schoolchildren, adolescents, nonwhite people, and individuals from the Southeast Region and Northeast Region (Table 2). There was a reduction in the proportion of patients admitted to the intensive care unit each year, as well as in the use of invasive mechanical ventilation. The total number and proportion of patients who died were highest in 2020. The highest number of children and adolescents with comorbidities was observed in 2020 (Table 3). The main comorbidities were asthma (42.57%), neuropathies (21.24%), and heart diseases (12.73%), followed by other pulmonary diseases (10.83%).

**Table 1:** Demographic and clinical characteristics of hospitalized children and adolescents in Brazil with acute respiratory failure from 2019 to 2022.

Characteristic	*N* ^a^	%
Socio demographics		
Gender		
Male	200,404	54.62
Female	166,500	45.38
Age		
Newborn	17,541	4.87
Infant	156,182	43.4
Preschool	82,936	23.05
School	54,839	15.24
Adolescent	48,355	13.44
Ethnicity		
White people	132,298	45.02
Black people	10,984	3.74
Yellow people	1,824	0.62
Brown people	146,763	49.94
Indigenous	2,025	0.69
Brazilian region^b^		
North	21,101	5.75
Northeast	72,264	19.68
Central West	31,514	8.58
Southeast	178,441	48.6
South	63,816	17.38
Clinical		
Symptoms		
Cough	282,569	83.76
Fever	243,919	74.83
Dyspnoea	214,599	69.31
Respiratory distress	209,767	68.56
O_2_ saturation <95%^c^	171,800	58.19
Vomiting	60,360	22.23
Pain throat	41,094	15.81
Fatigue	37,339	16.41
Diarrhoea	36,874	13.88
Abdominal pain	20,772	9.27
Anosmia	3,698	1.72
Ageusia	3,684	1.72
Comorbidities		
One	31,149	10.12
2 or more	8,454	2.3
Aetiology		
Influenza virus	7,784	2.12
Respiratory syncytial virus	32,453	8.84
SARS-CoV-2 virus	36,589	9.97
Other aetiologies^d^	290,310	79.07
Viral coinfection**	1,653	0.45
Outcomes		
Intensive care unit	80,658	24.63
Invasive ventilation	27,741	8.87
death	9,140	2.64

^a^ Number of individuals.

^b^ Brazilian region of residence.

^c^ Measured by pulse oximetry.

^d^ Other aetiologies: corresponds to the sum of other aetiological agents and cases without identified aetiology; SARS-CoV-2 = acute respiratory syndrome caused by coronavirus 2.

**Viral coinfection: corresponding to the presence of infection in the same patient with more than one of the viruses, influenza, respiratory syncytial virus or SARS-CoV-2 virus.

**Table 2:** Associations between demographic characteristics and acute respiratory failure incidence according to year of occurrence among hospitalized children and adolescents in Brazil.

	2019		2020		2021		2022		*p*	*p*	*p*
	*n* ^a^	%	*n* ^a^	%	*n* ^a^	%	*n* ^a^	%	2019 × 2020^b^	2019 × 2021^b^	2019 × 2022^b^
Gender											
Male	14,310	55.3	40,569	54.1	62,053	54.5	79,307	54.8	<0.01	<0.05	NS
Female	11,592	44.8	34,475	45.9	54,848	45.5	65,348	45.2	<0.01	<0.05	NS
Age											
Newborn	1,275	4.94	4,145	5.53	6,228	5.47	5,874	4.06	<0.01	<0.01	<0.01
Infant	16,099	62.3	24,210	32.3	48,386	42.5	67,048	46.4	<0.01	<0.01	<0.01
Preschool	4,236	16.4	15,381	20.5	26,376	23.2	36,801	25.5	<0.01	<0.01	<0.01
School	2,414	9.35	14,156	18.9	15,983	14.1	22,207	15.4	<0.01	<0.01	<0.01
Adolescent	1,805	6.99	17,012	22.7	16,799	14.8	12,680	8.77	<0.01	<0.01	<0.01
Ethnicity											
White People	10,975	51.3	24,424	40.6	39,408	43.5	55,763	48.3	<0.01	<0.01	<0.01
Black People	778	3.64	2,832	4.71	3,801	4.19	3,387	2.93	<0.01	<0.01	<0.01
Yellow People	102	0.48	407	0.68	560	0.62	695	0.6	<0.01	<0.01	<0.01
Brown People	9,241	43.2	31,941	53.1	46,448	51.2	55,067	47.7	<0.01	<0.01	<0.01
Indigenous	302	1.41	516	0.86	490	0.54	658	0.57	<0.01	<0.01	<0.01
Brazilian Region^c^											
North	2,668	10.3	5,995	7.98	5,500	4.83	6,020	4.16	<0.01	<0.01	<0.01
Northeast	4,668	18	17,898	23.8	22,440	19.7	24,310	16.8	<0.01	<0.01	<0.01
Central West	2,995	11.6	5,346	7.12	8,440	7.4	14,253	9.85	<0.01	<0.01	<0.01
Southeast	8,855	34.2	37,229	49.6	60,025	52.7	69,827	48.3	<0.01	<0.01	<0.01
South	6,723	26	8,661	11.5	17,579	15.4	30,289	20.9	<0.01	<0.01	<0.01

^a^ Number of hospitalized individuals.

^b^ Each year was compared to 2019; NS, nonsignificant.

^c^ Brazilian region of residence.

**Table 3:** Associations between clinical characteristics and the aetiology of acute respiratory failure in hospitalized children and adolescents in Brazil according to the year of occurrence.

	2019		2020		2021		2022		*P*	*P*	*P*
	*N* ^a^	%	*N* ^a^	%	*N* ^a^	%	*N* ^a^	%	2019 × 2020	2019 × 2021	2019 × 2022
Outcomes											
Hospitalization in ICU^b^	8,094	31.9	18,005	26.5	23,870	23.9	29,506	23	<0.01	<0.01	<0.01
Invasive ventilation	3,960	16	6,526	10.1	7,521	7.9	9,270	7.6	<0.01	<0.01	<0.01
Death	914	3.8	3,001	4.3	2,821	2.7	2,215	1.6	<0.01	<0.01	<0.01
Etiologies									<0.01	<0.01	<0.01
Influenza^c^	2,258	8.7	710	1	1.272	1.1	3,437	2.4	<0.01	<0.01	<0.01
RSV^d^	5,103	20	1,081	1.4	9.659	8.5	15,558	11	<0.01	<0.01	<0.01
SARS-CoV-2^e^	0	0	8,592	11.4	10,601	9.3	15.416	11	<0.01	<0.01	<0.01
Coinfections^f^	93	0.4	85	0.1	593	0.5	851	0.7	<0.01	<0.01	<0.01
Other aetiologies^g^	18,455	71.2	64,661	86.1	91,859	80.6	109,437	76	<0.01	<0.01	<0.01
Comorbidity	3,815	14.2	14,142	16.9	14,715	10.6	15,382	9.7	<0.01	<0.01	<0.01

^a^ *N*, number of individuals hospitalized by aetiology and with comorbidities.

^b^ ICU, intensive care unit.

^c^ Influenza virus infection.

^d^ Respiratory syncytial virus infection.

^e^ SARS-CoV-2 = acute respiratory syndrome caused by coronavirus 2.

^f^ Coinfections: corresponding to the presence of infection in the same patient with more than one of the viruses, influenza, RSV or SARS-CoV-2.

^g^ Other aetiologies: corresponding to the sum of other aetiological agents and cases without identified aetiology.

Each year was compared to 2019.

### Aetiology of acute respiratory failure

**Figure 1:**
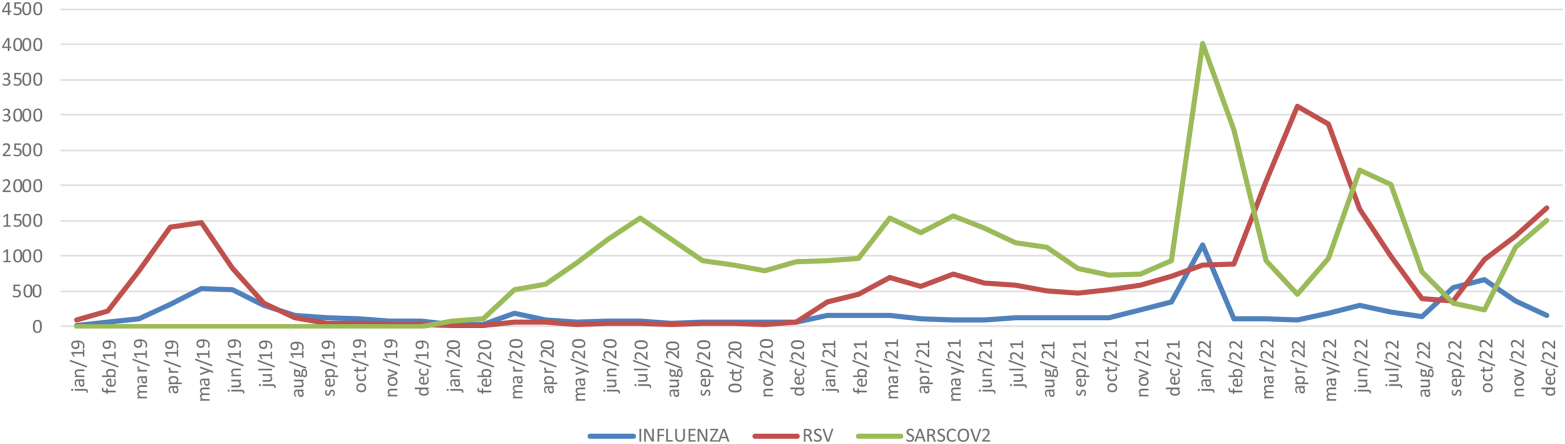
Seasonality of acute respiratory failure due to influenza virus, respiratory syncytial virus, or SARS-CoV-2 from 2019 to 2022 in hospitalized children and adolescents in Brazil. INFLUENZA = number of influenza virus infections per month; RSV = number of respiratory syncytial viruses per month; SARS-CoV-2 = number of acute respiratory syndromes caused by coronavirus 2 per month.

In 2019, the RSV season began in February/March, with a peak in May, and the influenza season occurred between March and April, with a peak in June/July. In February 2020, SARS-CoV-2 emerged, with a peak occurring in July. During 2020, there was a significant reduction in the number of patients with ARF with RSV and influenza. In 2021, there was a resurgence of RSV infection in January, and the incidence of RSV remained stable throughout the year, with no other peak. In this year, there was an increase in the number of hospitalized patients infected with SARS-CoV-2 in March and May. In January 2022, there was a significant increase in the incidence of ARF due to influenza, RSV and SARS-CoV-2 infections. The peak number of RSV infections occurred in April and May, and a new increase in RSV and SARS-CoV-2 infections occurred at the end of 2022 (Figure 1).

### Characteristics of patients with infection caused by RSV

**Figure 2:**
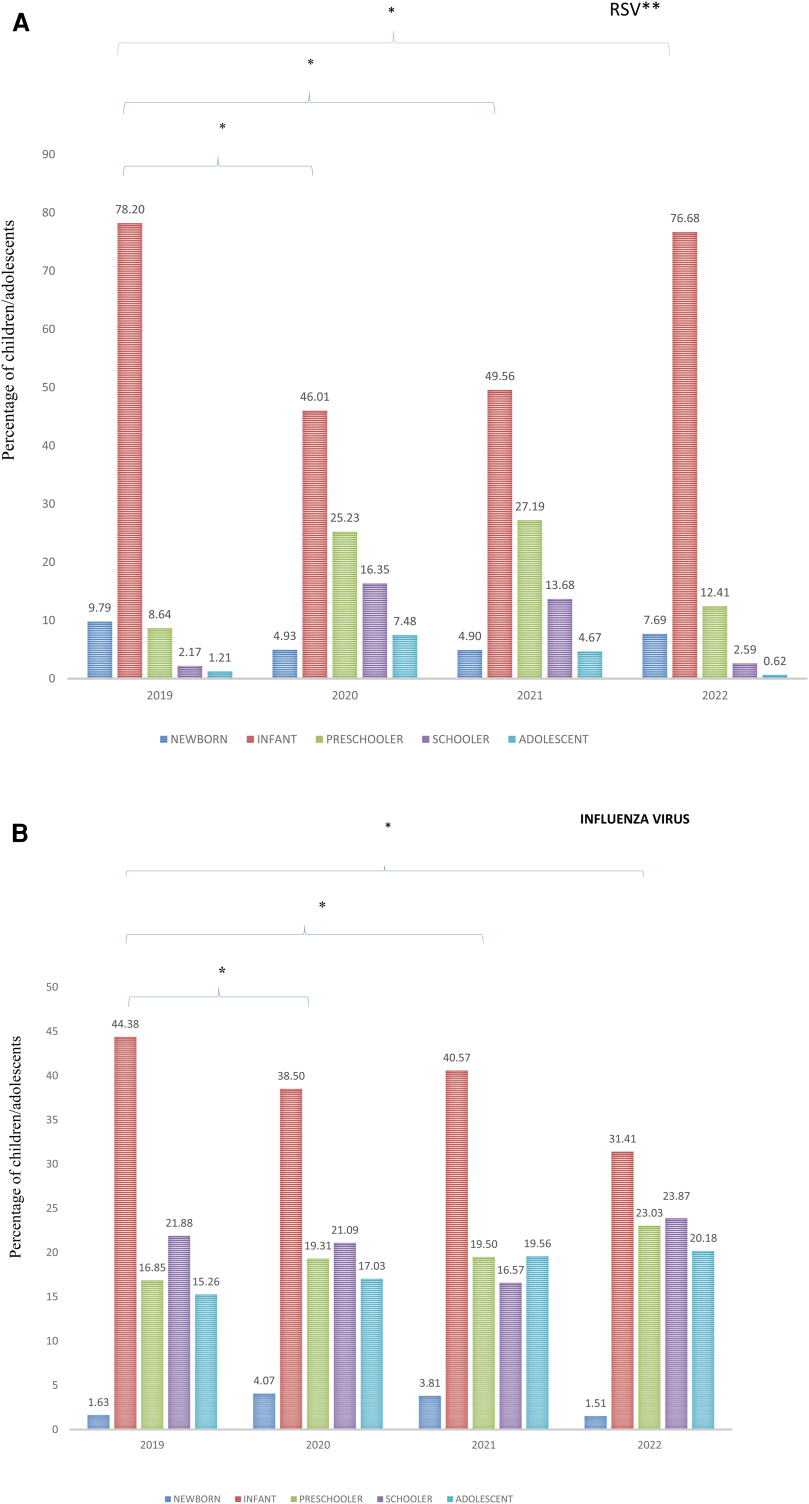
**(A)** Percentage of hospitalized children and adolescents with acute respiratory failure and respiratory syncytial virus infection in Brazil according to age range from 2019 to 2022. *p < 0.01; each year is compared to 2019; **RSV = respiratory syncytial virus. **(B)** Percentage of hospitalized children and adolescents with acute respiratory failure and influenza infection in Brazil, according to age range from 2019 to 2022. *p < 0.01; each year is compared to 2019.

In 2020, there was a reduction in the percentage of infants and newborns and a significant increase in the percentage of preschoolers, schoolchildren, and adolescents among patients with ARF with RSV. The mean age of patients with an RSV infection increased from 2019 to 2022 (consecutively: 1.1, 3.3, 2.9, and 2.8 years). There were fewer RSV infections in 2022 than in 2019, but the proportion of cases in infants was similar to that in 2019 (Figure 2A). Among individuals with an RSV infection, there was an increase in the proportion of individuals with comorbidities in 2020; however, in 2022, this proportion was lower than that in 2019 (Table 4).

**Table 4:** Presence of comorbidities according to aetiology of acute respiratory failure in children and adolescents from 2019 to 2022.

	Comorbidity	2019	2020	2021	2022	*P* ^#^	*p* ^#^	*p* ^#^
						2019 × 2020	2019 × 2021	2019 × 2022
Etiology								
Influenza	*N* ^a^	410	115	167	500	NS	*p* < 0.01	*p* < 0.01
	%	17.9	15.5	11	13.8			
RSV	*N* ^a^	468	170	997	712	*p* < 0.01	NS	*p* < 0.01
	%	9	14.7	9.3	4.4			
SARS-CoV-2	*N* ^a^		1,561	1,380	1,933	*p* < 0.01	*p* < 0.01	*p* < 0.01
	%		17	11.8	12			
Other aetiologies^b^	*N* ^a^	2,937	12,296	12,171	12,237	*p* < 0.01	*p* < 0.01	*p* < 0.01
	%	15.2	17	10.6	10			

^a^ *N*, number of individuals with comorbidity; NS, nonsignificant.

^b^ Other aetiologies: corresponding to the sum of other aetiological agents and cases without identified aetiology; Influenza = influenza virus infection; RSV = respiratory syncytial virus infection; SARS-CoV-2 = acute respiratory syndrome caused by coronavirus 2.

^#^ Each year was compared to 2019.

### Characteristics of patients with infection caused by influenza virus

There was an increase in the percentage of infected preschoolers and adolescents each year. In 2020 and 2021, the percentage of infected newborns also increased. The highest percentage of schoolchildren with influenza was observed in 2022 (Figure 2B). The mean age of individuals affected by influenza increased from 2019 to 2022 (consecutively: 4.8, 5.0, 5.0 and 5.6 years) (Table 3). The proportion of patients with comorbidities was lower in 2021 and 2022 than in 2019 (Table 4).

### Severety of acute respiratory failure

Multivariate analysis of the associations between the aetiology and severity of ARF in each study year was adjusted for gender, age, ethnicity (white or nonwhite people), origin, and comorbidities.

In all the years studied, the older the patient was, the lower the odds of ICU admission, but the greater the odds of death. There were no significant differences in the odds of ICU admission between males and females, except in 2021, when females had 3% higher odds than males did. The chances of death were similar regardless of gender.

Nonwhite people had significantly lower odds of ICU admission and greater odds of death in all years studied. Individuals from North Brazil and Northeast Brazil had lower odds of ICU admission after 2020 and greater odds of death in all years. The presence of comorbidities increased the risk of ICU admission in all years studied, as well as the odds of death, except in 2021 (Table 5).

**Table 5:** Multivariate analysis of the associations between aetiology and severity outcomes of acute respiratory failure according to year of occurrence, adjusted for gender, age, ethnicity, origin, and comorbidities, in children and adolescents in Brazil from 2019 to 2022.

		2019				2020				2021				2022			
		OR	*P*	IC 95%		OR	*P*	IC 95%		OR	*P*	IC 95%		OR	*P*	IC 95%	
ICU	Other aetiologies^d^	1.00				1.00				1.00				1.00			
	Influenza^a^	0.83	0.003	0.73	0.94	1.03	0.786	0.84	1.26	0.97	0.738	0.84	1.14	1.16	0.004	1.05	1.29
	RSV^b^	1.10	0.033	1.01	1.20	1.10	0.244	0.94	1.30	0.90	0.001	0.84	0.95	1.49	<0.001	1.41	1.56
	SARS-CoV-2^c^					1.08	0.011	1.02	1.15	1.19	<0.001	1.12	1.26	1.32	<0.001	1.26	1.39
	Gender^e^	1.02	0.471	0.96	1.10	1.02	0.285	0.98	1.06	1.03	0.04	1.00	1.07	1.02	0.214	0.99	1.05
	Age^f^	0.86	<0.001	0.83	0.89	0.84	<0.001	0.83	0.85	0.85	<0.001	0.84	0.86	0.88	<0.001	0.86	0.89
	Nonwhite^i^	0.91	0.017	0.83	0.98	0.85	<0.001	0.81	0.89	0.83	<0.001	0.79	0.86	0.80	<0.001	0.77	0.83
	North^g^	0.87	0.545	0.54	1.38	0.61	<0.001	0.48	0.78	0.63	0.002	0.48	0.84	0.62	0.005	0.44	0.87
	Comorbidity^h^	1.74	<0.001	1.62	1.87	1.46	<0.001	1.40	1.51	1.64	<0.001	1.57	1.70	1.48	<0.001	1.42	1.54
Death	Other aetiologies^d^	1.00				1.00				1.00				1.00			
	Influenza^a^	1.32	0.016	1.05	1.66	1.30	0.209	0.86	1.94	1.60	0.007	1.14	2.25	1.28	0.096	0.96	1.71
	RSV^a^	0.70	0.002	0.56	0.87	0.73	0.15	0.47	1.12	0.64	<0.001	0.52	0.78	0.82	0.05	0.67	1.00
	SARS-CoV-2^c^					2.41	<0.001	2.18	2.66	3.24	<0.001	2.94	3.57	3.01	<0.001	2.69	3.36
	Gender^e^	1.10	0.202	0.95	1.28	1.07	0.123	0.98	1.16	1.02	0.654	0.94	1.11	1.06	0.197	0.97	1.17
	Age^f^	1.15	<0.001	1.08	1.24	1.11	<0.001	1.07	1.14	1.25	<0.001	1.21	1.29	1.03	0.129	0.99	1.08
	Nonwhite^i^	1.22	0.033	1.02	1.46	1.04	0.397	0.95	1.14	0.90	0.026	0.82	0.99	1.09	0.156	0.97	1.24
	North^g^	2.40	<0.001	1.70	3.38	1.00	0.93	0.90	1.10	1.08	0.157	0.97	1.19	3.07	<0.001	2.36	3.99
	Comorbidity^h^	1.94	<0.001	1.71	2.21	1.03	0.472	0.95	1.12	1.01	0.795	0.91	1.12	2.35	<0.001	2.15	2.57

ICU, intensive care unit admission; OR, odds ratios obtained via the logistic regression model.

^a^ Influenza virus infection.

^b^ Respiratory syncytial virus infection.

^c^ SARS-CoV-2, acute respiratory syndrome caused by coronavirus 2.

^d^ Other aetiologies: corresponding to the sum of other aetiological agents and cases without identified aetiology.

^e^ Gender reference group = male.

^f^ Age: category variable treated as continuous.

^g^ Brazilian region of residence.

^h^ Presence of one or more comorbidities.

^i^ Nonwhite includes brown people, black people, yellow people and indigineous.

In 2019, influenza infection was associated with lower odds of ICU admission but with greater odds of death. However, RSV infection was associated with increased odds of ICU admission and decreased odds of death. In 2020, SARS-CoV-2 infection was identified as a significant risk factor for mortality and increased the odds of ICU admission and death. In this year, the associations of ICU admission and death with influenza and RSV did not reach statistical significance. In 2021, influenza virus infection was associated with increased odds of death, and RSV infection was associated with decreased odds of ICU admission and death. In 2022, influenza and RSV infections were associated with greater odds of ICU admission. Influenza was not associated with increased odds of death in 2022 (Table 5).

## Discussion

After the onset of the COVID-19 pandemic in 2020, there was a significant increase in the number of children and adolescents hospitalized with ARF in Brazil. This increase was progressive until 2022 and included changes in the characteristics of the affected individuals. There was a reduction in the proportion of infected infants and an increase in the proportions of infected preschoolers, schoolchildren, and infected adolescents. The incidence of ARF caused by RSV and influenza infections decreased dramatically during the pandemic, with a resurgence in 2021.

Among individuals aged between 0 and 20 years, the highest proportion of older children and adolescents with ARF during the COVID-19 pandemic was consistent with the findings of other studies that reported fewer SARS-CoV-2 infections in infants, which differs from the typical characteristics of other respiratory viruses, such as RSV, for which more severe cases occur in early life (16–19). We do not believe that the greater increase in the number of schoolchildren and adolescents than in the number of infants hospitalized from 2020 to 2021 was due to the characteristics of the social distancing measures adopted, since in Brazil, schools remained closed during the same period as nurseries and kindergartens during the COVID-19 pandemic. However, it is possible that older children, especially adolescents, have followed social distancing guidelines less rigorously than young children, who have less autonomy and remain under the care of their guardians.

The pathophysiological mechanisms that lead to this protection in younger children are not yet fully understood. It is possible that characteristics of the immune response, such as the predominance of innate immunity, especially natural killer cells, and the development of immunological memory in response to other respiratory infectious processes, may impact a child's ability to respond to SARS-CoV-2 infection, leading to decreased severity (20). The presence of more B and T lymphocytes in younger children could also prevent excessive inflammatory reactions, reducing the risk of complications (21). Other hypotheses emphasize that children exposed to seasonal coronavirus infections may develop cross-immunity to more severe infections, in addition to constant immune training resulting from frequent exposure to vaccines during early childhood, which acts as a protective factor (22). Studies investigating the factors involved in viral binding to the epithelium responsible for the entry of the virus into host cells have shown distinct characteristics between children and adults, although the subject is still a matter of controversy. The main known factors include angiotensin-converting enzyme 2 (ACE2), transmembrane serine protease enzyme 2 (TRMPRSS2), furin protease, and cathepsin. Bunyavanich et al. (2020) reported lower expression of ACE2 in the nasal epithelium of children than in that of adults (23). In agreement with these findings, an analysis of different SARS-CoV-2 strains performed by Zhu et al. demonstrated that the ancestral lineages of SARS-CoV-2 replicate less in nasal epithelial cells at the air–liquid interface in children than in adults. However, this pattern was not observed for the Omicron variant (24). However, some studies have reported similar levels of ACE2, Furin protease, TMPRSS2, and cathepsin in the upper airways of children and adults (25).

The greater incidence of ARF in older children and adolescents may also be due to the increase in the age of individuals affected by RSV and influenza (26). Our results show that at the beginning of the pandemic, infections with these viruses were more common among older individuals, which may also be associated with a high prevalence of comorbidities in these individuals.

The reduction in the incidence of infections caused by RSV and influenza in 2020 was compatible with the implementation of protective measures, such as the use of face masks, alcohol gel, social distancing and school closures, adopted in the country beginning in March of that year (27). In fact, protective measures against the transmission of respiratory viruses during pandemics in several countries have been studied. A retrospective, multicentre observational study that included 22 emergency departments in cities in Italy reported a 97% decrease in the number of RSV diagnoses between 2019 and 2020 (28). Our results revealed a decrease of 79%.

In agreement with this hypothesis, there was an increase in respiratory virus infections in 2022 compared with 2019, suggesting that respiratory virus infections occurred in a greater number of susceptible children in 2021, as protection restrictions were relaxed (29). The lack of immune stimulation by respiratory viruses over a prolonged period may have increased the number of immunologically vulnerable individuals. Studies conducted in Canada and the Netherlands have shown that neutralizing antibody titres against RSV are reduced in infants and women of childbearing age. These data suggest that the vertical transmission of antibodies may have decreased with an increase in the number of immunologically vulnerable infants, in addition to the absence of repeated exposure in the following years, favouring a larger population of older children who had not been exposed to RSV in their first year of life (30, 31). Our results revealed that the mean age of children with RSV-related ARF increased from 1 to 3 years of age from 2019 to 2021.

A 72% reduction in the total number of ARF cases due to influenza was also observed from 2019 to 2020. These results agree with what has been observed in other countries. Olsen et al. reported the behaviour of influenza infections in the USA, Australia, Chile and South Africa. After the adoption of measures to reduce SARS-CoV-2 transmission, the percentage of positive samples in the USA decreased from more than 20% to 2.3%. Low virus activity also occurred in countries in the Southern Hemisphere. The decrease in influenza circulation occurred shortly after the implementation of measures to limit the transmission of SARS-CoV-2. These two viruses are transmitted primarily through droplets, but the transmissibility of seasonal influenza is lower than that of other coronaviruses, which may have contributed greatly to the reduction in the number of influenza infections during this period (32).

Interestingly, after resurgence, the increase in the proportion of individuals with RSV infections was more pronounced than that of individuals with influenza infections, except in January 2022, when there was an influenza A (H3N2) epidemic in Brazil. It is possible that the immunization of mothers and children older than 6 months with the influenza vaccine conferred additional protection to children and adolescents; however, no vaccines against RSV were available during this period. In 2021, influenza infection was associated with a greater risk of death, and RSV infection was associated with a lower risk of death in 2021 than in 2019. In 2022, RSV infections led to an even greater increase in the odds of ICU admission but a decrease in the odds of death, maintaining the clinical characteristics expected for this infection (33).

The pandemic has also affected the seasonality of respiratory viral infections. The largest number of reported cases occurred in the southeastern region of Brazil, where the RSV season before the pandemic was characterized by the onset of infections in February/March and a peak in April/May. In 2021, infections occurred throughout the year, with a peak in November/December, a peak in April/May, and a new peak in October 2022. Changes in seasonality patterns have also been described in other regions of the world (34, 35).

In addition to a higher incidence, our results indicate a greater severity of infection in older children and adolescents and in those with SARS-CoV-2 infection, who had greater odds of death. Previous studies have suggested differences between children and adults in terms of pulmonary infection severity. Reduced and epithelial cell-specific ACE2 expression was observed in lung samples from developing mice. Similarly, TMPRSS2 expression was initially lower in the lungs of developing mice but increased over time, following a pattern similar to that observed in humans, where adults have higher levels than children do (36).

Aetiology was an important factor affecting the severity of ARF. Before the pandemic, the highest odds of death were associated with influenza infection, but after the emergence of SARS-CoV-2, this virus became the main factor associated with greater odds of death, even at the end of the pandemic (37). With the resurgence of RSV and influenza, patients with these infections had greater odds of ICU admission than did those with infections in 2019 did, suggesting greater severity of these postpandemic cases but no greater odds of death (8, 26, 38).

During the years studied, the presence of comorbidities such as chronic lung disease and heart disease, among others, resulted in a significant increase in the number of ICU admissions among patients with ARF. Paediatric patients with comorbidities who contract SARS-CoV-2 have a greater likelihood of complications and unfavourable outcomes, and this likelihood increases with age (39).

Other factors associated with greater odds of death were residence in the North/Northeast region and greater disease severity in nonwhite people. These findings may be associated with social characteristics in Brazil, as access to health care in highly complex services is still greater for white people with greater purchasing power (40). This inequality may have been more important at the beginning of the pandemic when there was an unexpected overload and unpreparedness of public services (41).

In the present study, the severity of ARF was best assessed by the odds of death. Hospitalization in the intensive care unit and the use of mechanical ventilation, which are usually markers of severity, exhibited different behaviours during the pandemic because they depended on the ability of health services to respond to the increased demand caused by the pandemic. The mortality rate in 2020 was greater than that in 2019, but despite the increase in the absolute number of patients, the proportion of patients treated with invasive mechanical ventilation was lower. Being nonwhite and residing in North Brazil or Northeast Brazil were associated with lower odds of ICU admission and higher odds of death before the pandemic, which was still true in 2022. These results also suggest the initial lack of preparedness of the health system for providing care during the pandemic, as there were lower odds of ICU admission and increased odds of death (42).

The associations found in the present study cannot be used to draw causal inferences because of the retrospective design, which included data collected from Brazil's national computer system. We were not able to analyse the use of passive immunization for RSV since the database does not present this information. However, as the number of premature babies was small (4697 of 367,135 individuals), the lack of this information did not interfere with the interpretation of the results.

## Conclusion

The COVID-19 pandemic has significantly influenced the prevalence, demographic characteristics, severity, and aetiology of ARF in children and adolescents. Older children, adolescents, and individuals with comorbidities were more vulnerable to SARS-CoV-2, RSV and influenza infections. There was a notable reduction in the prevalence and severity of RSV and influenza infections during the pandemic. After this reduction, a resurgence with an out-of-season pattern was observed for both, but with similar odds of death to those in the prepandemic year. Understanding the impact of the COVID-19 pandemic on the dynamics of the main ARF agents in children and adolescents will contribute to the development of pharmacological prophylactic measures, the efficient implementation of nonpharmacological measures and the preparation of the health system to address the effects of viral respiratory infections.

## Data Availability

The datasets presented in this study can be found in online repositories. The names of the repository/repositories and accession number(s) can be found in the article/Supplementary Material.
